# Participant and group facilitator perspectives on a novel culturally tailored diabetes self-management program for African Americans

**DOI:** 10.21203/rs.3.rs-3136363/v1

**Published:** 2023-07-18

**Authors:** Adati Tarfa, Ejura Salihu, Phanary Xiong, Cierra Brewer, Martha Maurer, Yao Liu, Olayinka Shiyanbola

**Affiliations:** University of Wisconsin School of Pharmacy Madison; University of Wisconsin School of Pharmacy Madison; University of Wisconsin School of Pharmacy Madison; University of Wisconsin School of Pharmacy Madison; University of Wisconsin School of Pharmacy Madison; University of Wisconsin; University of Wisconsin School of Pharmacy Madison

**Keywords:** African Americans, diabetes self-management, peer support, community engagement

## Abstract

**Background:**

African Americans with type 2 diabetes experience disparities in their care and diabetes health-related outcomes. Diabetes self-management programs such as Healthy Living with Diabetes (HLWD) are important but do not account for the unique cultural experiences of African Americans. As well, a culturally tailored program focused on addressing sociocultural beliefs and providing race-congruent peer support, Peers LEAD (Peers Supporting Health Literacy, Self-Efficacy, Self-Advocacy, and Adherence) was implemented in two midwestern cities to improve medication adherence but does not include diabetes self-management topics included in HLWD. In attempt to fill the gaps from both HLWD and Peers LEAD, Peers EXCEL (Peers’ Experience in Communicating and Engaging in Healthy Living) was designed to integrate both programs.

**Methods:**

Our study explored the perceptions of African American participants and facilitators of the separate HLWD and Peers LEAD programs, on the proposed new Peers EXCEL program using focus groups and interviews. Findings were analyzed by research assistants trained in qualitative research using deductive and inductive open coding approaches.

**Results:**

Participants described the lack of cultural fit of the current HLWD program for African American communities and proposed strategies to enhance Peers EXCEL’s impact in African American communities. They shared the need to include topics such as the relationships between systemic racism and diabetes.

**Conclusion:**

Participants’ feedback of Peers EXCEL reveals the importance of including various community member perspectives in the design of new diabetes self-management programs tailored for African Americans.

## Background

African Americans are a historically marginalized population bearing substantial health disparities in the United States (1). Compared to non-Hispanic whites, African Americans are twice as likely to be diagnosed with type 2 diabetes and two to four times more likely to experience diabetes-related complications such as kidney failure, blindness, lower-limb amputations, and amputation-related mortalities (2). Disparities in diabetes diagnoses and prognoses between non-Hispanic whites and African Americans are associated with multiple factors, including lower health literacy (3), greater distrust of the health system and healthcare providers (4), and more negative health beliefs (5). Thus, there is a critical need for culturally tailored programs that address these factors associated with diabetes health disparities among African Americans.

We previously developed a peer-led intervention to specifically address health beliefs and increase medication adherence for improving diabetes-self management among African Americans, the Peers LEAD (Peers Supporting Health Literacy, Self-Efficacy, Self-Advocacy, and Adherence) program, which was found to be both feasible and acceptable by African Americans receiving peer support. In addition to providing one-on-one peer support, this intervention also includes group sessions addressing health beliefs and improving communication with healthcare providers. However, there are other important aspects of diabetes self-management, such as addressing diet and exercise, that were not included in the Peers LEAD program.

To address gaps in these other aspects of diabetes self-management, we proposed to combine Peers LEAD with a well-established, community-based diabetes self-management program, Healthy Living with Diabetes (HLWD) (6) (7). While HLWD is an evidence-based program, it has not been culturally tailored for African American populations. Notably, African Americans have been found to be less likely to participate in or complete the HLWD program (8) and addressing diabetes beliefs and knowledge using culturally tailored diabetes self-management interventions has been shown to produce positive health outcomes in African Americans (9, 10). This proposed program, Peers EXCEL (Peers’ Experience in Communicating and Engaging in Healthy Living), seeks to improve diabetes self-management among African Americans by combining key features of Peers LEAD and HLWD programs that complement one another. We sought to work collaboratively with African American stakeholders in developing and implementing interventions since this approach can improve their cultural relevance and subsequently, may increase their impact on reducing health disparities. In a prior work, we described perspectives of Peers EXCEL from healthcare professionals and organizational leaders serving African American communities and/or providing diabetes education. In this study, we engaged African Americans with type 2 diabetes who previously participated in either Peers LEAD or HLWD, as well as Peers LEAD peer supporters and HLWD facilitators, to obtain their perspectives on the design and implementation of Peers EXCEL to provide culturally tailored diabetes self-management.

## Methods

### Description of the Peers EXCEL program

**Figure 1:**
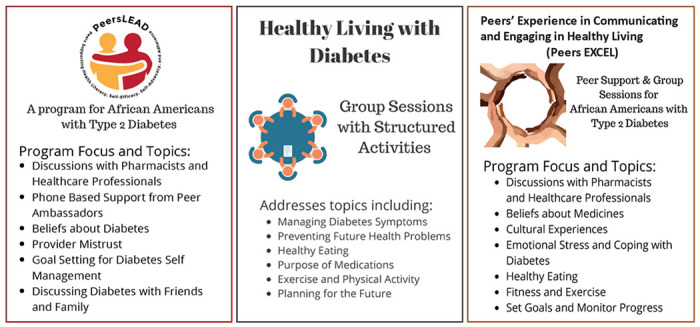
Description of diabetes self-management programs shared with study participants

Peers EXCEL is a novel diabetes self-management program that integrates the culturally tailored Peers LEAD program for African Americans with topics from HLWD, a community-based diabetes self-management program (Figure 1) while also providing the one-on-one peer-support format of the Peers LEAD program. In Peers EXCEL African Americans with diabetes who self-report poor medication adherence are paired with other African Americans with diabetes who have high medication adherence. Participants then meet during one-on-one peer support phone calls and attend group education sessions. Six weeks of group education sessions are led by a trained African American facilitator, focusing on diet and exercise, goal setting, stress management, etc. A pharmacist and a healthcare provider lead two separate group education sessions to address beliefs and misperceptions about medicines and diabetes, including enhancing provider communication. Participants are also offered the opportunity to connect with a Community Health Worker (CHW) during the program.

### Participant Recruitment

We recruited English-speaking adults 30-65 years of age with type 2 diabetes who self-identify as Black/African American and previously participated in either the Peers LEAD or HLWD programs. We used records of participants from these programs and personal contact by community partners to facilitate recruitment including word of mouth, phone calls, and emails. All participants provided verbal informed consent. All research activities were reviewed by the (University name) Institutional Review Boards (IRB) staff, which deemed the study exempt due to being interview and focus group research. The study was conducted in accordance with the Declaration of Helsinki.

### Data Collection

#### Individual Interviews with Peers LEAD and HLWD Participants

Interviews were conducted by the research team members, who were trained in conducting qualitative interviews by the Principal Investigator (P.I.) (initials). The researchers conducted 30–45-minute semi-structured, individual interviews with Peers LEAD and HLWD participants. The interviews occurred via a secure web-based meeting platform (WebEx, Cisco Systems, San Jose, CA, USA) from September 2020 to December 2020. An interview guide was developed based on three domains of the Consolidated Framework for Implementation Research (CFIR): (1) intervention characteristics (e.g., relative advantage, complexity), (2) outer setting (e.g., individual needs, barriers), and (3) inner settings (e.g., compatibility). Interviewers provided participants with figures describing the topics and focus of the separate Peers LEAD and HLWD programs, and the integrated program, Peers EXCEL (Figure 1).

Individual interviews were used because they are ideal for gathering in-depth views about a particular topic in a safe environment and allow researchers to delve deeply into delicate issues that participants may otherwise be uncomfortable sharing in a large group (11). The interview guide included open-ended questions that allowed participants to share their in-depth perspectives of Peers EXCEL. Interviews were audio-recorded and transcribed verbatim by a professional transcriptionist with any identifying information redacted. Interview participants received a $50 stipend.

#### Focus Group Meetings with Peers LEAD peer supporters and HLWD facilitators

We conducted two separate focus group meetings with past Peers LEAD peer supporters, who lead the one-on-one peer support phone calls, and HLWD facilitators, who led group sessions for HLWD participants. Each 90-minute meeting was hosted via a secure web-based meeting platform (WebEx, Cisco Systems, Inc., San Jose, CA) and the meetings were conducted from October 2020 to December 2020. The P.I. (initials), an experienced focus group facilitator, an academic pharmacist, and a qualitative researcher, moderated the sessions. The focus group moderator was supported by research team members, who assisted with taking notes and managing the virtual platform. Focus group participants were provided with figures describing the topics and focus of the Peers LEAD, HLWD, and Peers EXCEL programs (Figure 1). Focus groups are complementary to individual interviews by expanding discussion upon group responses (12). The focus group guide contained open-ended questions that allowed participants to share their in-depth perspectives on combining the Peers LEAD and HLWD programs. Focus group discussions were audio-recorded and transcribed verbatim by a professional transcriptionist with all identifying information redacted. Focus group participants received a $50 stipend.

### Data Analysis

Research assistants trained in qualitative research (health services research graduate students: (initials) and pharmacy professional students: (initials)) used deductive and inductive open coding approaches to conduct a directed content analysis (13) of the interviews and focus groups transcripts. To establish rigor of the data, research assistants focused on two criteria: (1) credibility and (2) confirmability. These criteria were achieved by having multiple research assistants code the transcripts independently and then met together with the entire research group to discuss similarities and differences to reach a consensus in interpreting the results.

Two research teams were formed to analyze the focus groups (initials) and interview (initials) transcripts independently. The first cycle of analysis consisted of creating a coding framework. The frameworks were finalized through an iterative review process with feedback from the P.I. and (initials). In the second analysis cycle, the analysis teams met independently to code and discussed their observations and findings. Differences in their findings were discussed among the analysis teams and shared with the entire research group until a consensus on data interpretation was reached. During the final analysis cycle, the analysis team met with O.S and M.M. to present findings and to fit codes from interviews and focus groups into higher-order categories. The interview and focus group data and coding methods were also reviewed by members of the (University Name) Institute for Clinical and Translational Research-Community Academic Partnership (ICTR-CAP) Qualitative Research Group. Data management was facilitated using NVivo software, Version 11.4.1 (QSR International, Melbourne, Australia).

## Results

Fourteen African Americans participated in the individual interviews and 19 African Americans participated in three separate focus groups to provide their perspectives on the combined Peers EXCEL program. The main themes were: (1) the lack of cultural fit of the HLWD program for African American communities, (2) proposed strategies to enhance Peers EXCEL’s impact in African American communities, and (3) important topics to cover in the Peers EXCEL program.

### Lack of cultural fit of the HLWD program for African American communities

#### How to deliver Peers EXCEL in African American communities

Participants perceived a lack of cultural fit between the delivery of HLWD content by facilitators who are not African American/Black (Table 1).

Their concerns included the lack of African American facilitators, and that HLWD facilitators lacked experience in working with African Americans to deliver the program in a culturally sensitive manner.

“I co-led [HLWD] with someone of another race [who is not Black]. And [HLWD participants] specifically requested for someone else that looked like me, basically, and looked like us, because majority of [HLWD participants] were Black…And [the non-Black HLWD co-facilitator], like they pulled me to the side after the meeting several times … would say things offensively, not necessarily intentional, but she just wasn’t, she didn’t have cultural humility.” – HLWD Facilitator #3

The contents and methods used to deliver HLWD were also areas of significant concern for HLWD participants. Participants perceived that the language used in the written script could have been better suited for an African American audience. HLWD facilitators also perceived that they had to read the HLWD script and could not adapt their delivery style to engage their audience more effectively.

“Just don’t see how anything from [the HLWD Program] is going to work [for Black participants] … I’m just saying, it was pretty much scripted, and you couldn’t veer off from the script … it’s hard to follow a script from Stanford [creators of HLWD] when you’re dealing with African Americans. That’s a whole, that’s like a different language.” – HLWD Participant #8

Participants recommended that when introducing the Peers EXCEL program to African Americans, an emphasis should be placed on the importance of the program and to avoid the appearance of disparaging or “talking down” to program participants.

“I think when [Black participants are introduced] to the [Peers EXCEL] program, it can’t be, ‘Well, we’re adding this because we know that, in the Black community, that you guys aren’t serious about taking your medication.’ It can’t be at a point of talking down to people … [Instead,] we’re doing this because we want to emphasize the importance.” Peers LEAD Participant #4

#### Advantages of Peers EXCEL relative to either the HLWD and Peers LEAD programs

Participants described how the Peers EXCEL format of group-based discussions and one-on-one peer mentorship pairings empower African Americans to ask questions, receive advice from peers, and encourage building connections between community members.

“And then when I joined this group, and I saw the fierceness of it and just listening to people talk about it, just really opened my eyes…I’m not afraid to ask questions now… So it opened me up to be a little more free to ask some questions from somebody else who gone through [living with diabetes] and to listen and get the advice from them.” – Peers LEAD Participant #6

A prominent feature of Peers EXCEL for participants is how it recognizes the disproportionate prevalence of diabetes in African American communities.

*“I think the program is much needed, is important. Usually, illness that strike the community, it strikes the African community the worst. We need to begin to focus on our community because there’s a need. Period.”* – Peers LEAD Participant #4

#### Perceptions of key topics, content, and format of Peers EXCEL

Participants noted having opportunities to interact with a healthcare provider in Peers EXCEL could improve communication between African Americans and their healthcare providers.

*“I just think that just having real conversations with African Americans about how important it is to have a primary care doctor, just something that simple. I know my family, a lot of them didn’t even have a primary care doctor.”* – Peers LEAD Participant #3

Participants emphasized the importance of trust in African American communities and how having a physician and pharmacist that African Americans can trust is an important part of the program.

“It seems like a couple things I think makes [Peers EXCEL] important, especially for the African American community…is trust. A lot of times, you’re not going to talk to your doctor or talk to individuals about things that are really causing you some issues, for whatever reason. And if you have an intimate connection with a doctor or a pharmacist or someone that you can trust to ask some of these questions, I think that would help as well with the community.” – Peers LEAD Participant #7

Peers LEAD participants perceived that having group meetings and receiving one-on-one phone calls from their peers made the program personal, which was viewed as a strength.

“The … program was very personal, one-on-one… But there’s something personal about working … one-on-one and knowing you’re going to get that [mentoring] phone call [from the Peers LEAD Ambassador] and then working with the entire group.” – Peers LEAD Participant #9

Participants shared how the Peers LEAD one-to-one peers support format was unique and that they appreciated being partnered. According to participants, an added benefit of the Peers LEAD one-on-one peer support is that having a partner makes them accountable for achieving their goals as they manage their diabetes.

“Peers LEAD was one-on-one. You actually had a one-on-one mentor. Very few are able to supply that. That one-on-one connection is nice.” – Peers LEAD Participant #8

“I think that [one-on-one peer support] would help because it would encourage the person to continue on with the fight. So with someone checking in constantly, I think it would push and motivate you to do your goals or succeed with your goals.” HLWD Participant #2

Participants described how HLWD content that focuses on goal setting would result in greater accountability regarding diabetes self-management when African Americans are provided with one-on-one peers support.

“I think that it really would be beneficial because…with the Healthy Living with Diabetes, we set goals, and we have our different things that we want to accomplish. I think if it was one-on-one, I got to be accountable, if [my Peers LEAD Ambassador] is … going to ask me, ‘Well, did you take your medication this morning?’ and ‘What was your blood sugar?’… That one-on-one makes it more accountable. Even if I don’t feel like doing it, I’m going to do it. I think that combining the programs … you would be more accountable” – HLWD Facilitator #5

### Strategies to improve Peers EXCEL to meet the unique needs of African Americans with diabetes

#### Broaden the reach of Peers EXCEL to African Americans

In addition to recognizing the need for a program focusing on diabetes in African American communities, participants shared recommendations on how the research team can enhance Peers EXCEL’s impact on African American communities (Table 2). These included broadening the program’s reach by including a wide range of individuals with different roles who could be involved in the recruitment. Additionally, participants perceived an opportunity for diabetes education to be tailored toward the diabetes self-management needs of young African Americans.

#### Engage highly motivated individuals in Peers EXCEL

Participants perceived that for individuals to participate in Peers EXCEL, they will need to be highly motivated to join a program that requires a long-time commitment over several weeks, and includes diabetes-related topics they may have not fully explored, such as exercising and cooking. Our participants were concerned that Peers EXCEL may be more limited in its success if recruited individuals lack sufficient motivation.

“That would be a long two-and-a-half hours to be working with [a participant] who don’t want to do better… especially when you start to … introduce exercise to individuals who haven’t exercised. You’re going to introduce new [ideas about] cooking to individuals who haven’t done [that type of] cooking. That may be a challenge, and I don’t want the program to fail because you don’t have certain individuals [who are at a sufficiently high] level of [motivation] to be a part of the program.” Peers LEAD Participant #5

Participants added that, to engage motivated individuals in the program, the recruitment process must be designed to ensure that participants are motivated to improve their health and are not participating solely to seek monetary incentives.

**“**I think the key is recruitment, and I remember [name of research team member] did an excellent job in… recruiting people and getting people. So the more thorough [the recruitment process,]… the better … You [should try] to weed out the people who just want the cash and [are] not really [motivated].” Peers LEAD Participant #5

#### How to encourage participation in Peers EXCEL

Some participants recommended that the research team use monetary incentives for recruitment into the Peers EXCEL program such as using vouchers that could be used towards purchasing healthy food or by providing gym memberships to promote positive lifestyle changes.

“Money is an incentive to get you there, which means once you get into that program, you might get motivated enough that you actually come out of there with something because it’s very informational if you’ve got diabetes. No telling, this program might just have turned somebody around…it might help save so many more.” – Peers LEAD Participant #6

“I think an incentive would be great, especially if the incentive also goes to purchasing food. Like for them to be a part of this, you’re talking about healthy eating, then they actually have vouchers to get these foods…if you’re talking about exercise…Maybe incentive to get back to the gym safely, that they have like a membership for six months or three months, or something like that, to actually get into gym or something like that… Because we’re talking about a lifestyle change.” – Peers LEAD Participant #1

Participants perceived that the recruitment process should be thorough in identifying individuals who are genuinely interested in improving their diabetes management and not only focused on the potential monetary incentives.

“I think the key is recruitment, recruiting people and getting people… You can kind of start to weed out the people who just want the cash and not really concerned.” – Peers LEAD Participant #5

Participants also shared how including interactive activities in Peers EXCEL could enhance trust and camaraderie within the Peers EXCEL program beyond discussing only diabetes-related topics.

“… a social outing and gathering and still having fun, doing something other than doing the diabetes thing, some fun activities, as well…they went out to eat together and did things together and took trips together… They can trust one another with what they had to say about the struggle of diabetes. And it’s just that camaraderie that helps outside of the thought of we’re just here for diabetes.” – Peers LEAD Participant #3

#### Topics to include in Peers EXCEL

Recommendations for additional topics to be addressed in Peers EXCEL include addressing the mental health issues experienced by those living with diabetes, and the historical context and impact of systemic racism on African Americans’ experiences of living with diabetes (Table 3).

Some participants shared personal experiences growing up in segregated environments and how that impacted their ability to lead healthy lives while living with diabetes.

“I know when we talked about the histories of some of our participants, we heard where they came from. Many of them came from segregated environments growing up… when they were younger, [they] would have been going the direction of leading a healthy life, but they had, of course, life issues that got in the way of eating…what’s considered to be a proper healthy diet.” – HLWD Facilitator #4

Participants discussed how the topics of HLWD such as diet and exercise will help them in achieving personal goals related to their diabetes self-management such as losing weight.

*“I think the more active components of Healthy Living with Diabetes would kind of help me with the active parts. Also, the dietary parts of it could help me to lose the weight I’ve been trying to lose for a while… [These] would be useful tools.”* HLWD Participant #3

Participants perceived the importance of the topics addressed in Peers EXCEL, especially stress management, which has a substantial impact on their ability to manage their diabetes.

*“I think [these topics are] very important to know because you can get stressed out, and you can get overwhelmed and forget about your health, and next thing you know, it cause a problem because you’re not taking care of yourself … stress is a big part of being a diabetic, to me.”* – Peers LEAD Participant #2

Participants were interested in discussing ways to decrease medication costs, and how to read nutrition labels. Other topics include setting personalized goals, with group-based discussions concerning goal setting.

I think if we could incorporate [goal setting] as a group instead of individuals, as [Peers LEAD] Ambassadors coming up with [those goals] … Speaking for my group, when we did talk about goals, some individuals didn’t think it was important to do. But [it might be more valued] if it’s a collective discussion and saying, part of the conversation is: we’re going to come up with goals.” – Peers LEAD Participant #7

## Discussion

Study participants perceived that the Peers EXCEL program combined the benefits of Peers LEAD and HLWD into a culturally adapted program that could effectively help African Americans with diabetes achieve their diabetes self-management goals. These participants shared the need for culturally adapting the HLWD program, strategies to enhance the impact of the Peers EXCEL program in African American communities, and important topics to cover in the Peers EXCEL program. Our participants also highlighted the importance of providing social support and building a trusting environment when engaging with African Americans by designing culturally appropriate programs.

A distinguishing feature of Peers EXCEL is the use of African American peer supporters and African American healthcare professionals to deliver the program. Culturally appropriate peer support programs engage members of the target community who understand the culture of the people, have an equal standing, have the same illness condition, and can connect with the group in a way acceptable to them (14). In addition, the shared experience of being African American has a powerful influence on how African American facilitators effectively approach their teaching by creating a supportive classroom environment where participants felt comfortable being honest in sharing their experiences, questions, and frustrations without the fear of judgment, condemnation, or ridicule(15). HLWD was a poor fit for African American communities because the facilitators were neither people of color nor had a history of working with African American communities. The HLWD program was perceived as scripted in its delivery which needed to allow for facilitators to tailor their delivery to fit its predominantly African American audience. An integral part of culturally appropriate programs is leveraging the cultural identities of the facilitators to adapt their assigned curricula so that the program will be more relevant to their African American participants (16).

We also found that our participants valued having trusted healthcare providers share their expertise within the Peers EXCEL program and that relationships between systemic racism and diabetes should be discussed. Mistrust of the healthcare system stemming from historical and current racism faced by African Americans significantly contributes to diabetes-related healthcare disparities (17). Mistrust was also identified as the most significant contributor to low rates of African American participation in diabetes self-management programs such as the HLWD (8). As such, it is important to note the significance of our participants’ outlook of Peers EXCEL as a program that could enhance trust between African American communities and pharmacists/healthcare providers. Furthermore, our participants recommended a discussion of the relationships between systemic racism and diabetes in Peers EXCEL, which is consistent with the finding that racism impacts diabetes beliefs among patients (18). Some African Americans perceive slavery as a factor that contributed to their development of diabetes (17, 19). Diabetes scholars support using Critical Race Theory (CRT) – inclusion of historicized and contextualized questions of race and racism – in health inequities research and diabetes self-management programs (20). Peers EXCEL has the potential to explicitly address the topic of systemic racism’s intersections with diabetes in the African American community.

In addition to the need to cover critical topic areas, our study participants recognized that a novel intervention such as Peers EXCEL would need rigorous recruitment strategies. Participants recommended the use of incentives related to program goals. For example, vouchers to purchase foods that promote healthy eating and gym membership to promote exercise. Studies have shown that individuals with diabetes are willing to consider financial incentives to improve diabetes self-management (21). Studies examining recruitment strategies of African Americans in a diabetes program showed that incentives are highly valued by participants (22, 23). However, our study participants also perceived that providing incentives for participating in the program should be complemented by using recruitment strategies that attract motivated participants to minimize the risk of high attrition rates during the program, given how challenging it can be to achieve goals set for diet and exercise.

### Limitations

In addition to its strengths, our study also had some limitations. While the CFIR framework was used to develop the focus group and interview guides, we found that participants’ responses did not adequately fit within the CFIR domains. Thus, we used an inductive approach to analyze the data. In addition, individuals who agreed to participate in interviews and focus groups may not represent all who participated in the Peers LEAD or HLWD programs.

## Conclusion

We conducted individual interviews and focus groups with African American stakeholders with prior experience in Peers LEAD and HLWD to further develop and refine the combined Peers EXCEL intervention. Interventions designed with the engagement of community members in a cultural context can improve health outcomes and potentially meet the unique needs of African Americans for diabetes self-management.

## Figures and Tables

**Table 1:** Theme: Perspectives on the lack of cultural fit of the Healthy Living with Diabetes (HLWD) program for African American communities

Subthemes	Quotes
Most current HLWD facilitators do not have a relationship with African American communities	*“A lot of times, [HLWD facilitators] don’t necessarily live in the [African American] community. And not only do they not live in the community, but they don’t even, have a relationship with the community, but they still facilitate things that affect the community … If you really want to help the community, you have to build relationships, and you really have to care about the community, and care about the people.” – HLWD Facilitator #7*
Lack of African American subject matter experts and facilitators in HLWD program	*“We had a nutritionist come in as a subject matter expert … and the [HLWD participants] wanted someone who looked like themselves. But at that time, we couldn’t find that individual. And so we still went ahead and did it because it was on the schedule, but I don’t think it matched up as well. It felt like the session was not the same as the other sessions.” – HLWD Facilitator #4*
Trust in and relatability of HLWD facilitators among African American participants	*“Not only was it that cultural context, that relatability to the participants, but it was also the age of the facilitator. I was always the youngest facilitator, and that was always a barrier in the beginning. [Participants] were like, ‘What you know about this? You only like 20 [years old].’ And I was like, well, I went through the class. I do know how to facilitate. But for at least three weeks, it was like, ‘No, we don’t really trust [this person] to really give us this information correctly’ …Age, especially in our community, equals wisdom when you’re facilitating this type of content.” – HLWD Facilitator #1*
Lack of cultural fit in HLWD program delivery methods	*“One of the challenges that I experienced was the participants did not like being read to… when you’re in the presence of people, you know, everything becomes conversational. The [participants] felt insulted by [reading to them]. And another thing was even just some of the language of the book, it was, for some participants, it was hard to read.” – HLWD Facilitator #3*

**Table 2:** Theme: Proposed strategies to enhance Peers EXCEL’s impact in African American communities

Subthemes	Quotes
Strategies to broaden the reach of the program by engaging a variety of people in different roles	*“Because it’s going to take a group of people to disseminate the information. There’s not enough people. I wish there was more of y’all to disseminate, educate our community. So we need people playing different roles at different levels to get the job done” – HLWD Participant #2*
Engage physicians to increase participation and partner with them in the program	*“…So maybe reaching out to some of the participants’ doctors and say, … ‘Can you share this information with some more of your [patients]?’… Somehow connect with the doctors and sort of be [working] hand in hand. That way [participants are] getting double support from your doctor and the peer person.” – Peers LEAD Participant #8*
Engage family members in the program	*“You know, like I said, it’s good sometimes to bring [participants’] mates because they’re not in it by themselves. If you’re living [with a] husband, wife, girlfriend, boyfriend, whatever, and are you the one cooking the meals? Bring [those people], open it up to family members”. – Peers LEAD Participant #8*
Engage older and younger people with diabetes differently by tailoring to their needs	*“Now the setting might have to be a little different because when you’re dealing with senior citizens with diabetes… you might have to change the dynamics to educating youth with diabetes… So if you have a young person, and you have a [Peers LEAD] Ambassador with them… [who is] African American… I think that they would be more readily [accepting of the program], and also understand that this isn’t a doom and gloom… you still have a quality of life. … and then you got some tools that you can use to maintain the qualities of life.” – HLWD Participant #3*
Ideas for physical locations to host the program	*“I think your program needs to be centralized in terms of having concrete building, a place where it’s designed for these programs to move forward. I mean, if I could go to a building that … has a big ol’ sign on the door, and I’m walking there, and there’s exercise equipment… [I’d think,] ’Oh, this is a unique place!’ … A place for diabetics to have a more healthy living and for them to interact.” – HLWD Participant #4* *“Instead of the [participants] coming to us, there has been situations where if they lived in a senior or a HUD housing, I would set up the meetings … if I got three or four people that said, ‘Hey, I want to do this.’ I would put out a flyer in the building and let them recruit … others in the building … People would walk by because it’s in that senior complex. They would say, ‘Well, what are you guys doing?’ And when we explain it, they’ll say, ‘Oh, I got diabetes’ or ‘My sister has diabetes, you know. Can I get in the class?’ … We would start a whole second group … Sometimes, we have to take the message to [the participants].” HLWD Facilitator #2*

**Table 3:** Theme: Perspectives on important topics to cover in Peers EXCEL

Subthemes	Quotes
Address mental health issues resulting from living with diabetes	*“[People] just think, [diabetes] is the end of my life as I know it. That’s how they look at it. And it’s not true. You can live with it. It’s manageable … They become very, very depressed. So I think having that addressed would be great.” – Peers LEAD Participant #11*
Discuss relationships between systemic racism and diabetes in the African American community	*“So it was not lost on our facilitators that, periodically, the question would come up, ‘Why are there just Black people in this class?’ It wasn’t specifically [limited to Black people] … Having conversations about the reality of… things like systemic racism, actually, is very relevant to this class because it’s part of the reason why [not everyone] would… have been receptive to certain individuals coming in and trying to facilitate the class.” – HLWD Facilitator #4*
Set reachable personalized goals	*“Making sure to emphasize that the goal can’t be so high where it’s going to frustrate you because you’re not reaching it or maintaining whatever you said your activity [goal] was going to be.” – HLWD Facilitator #6*
Provide information on how to reduce medication cost	*“There are things that we can do to help bring down the exorbitant costs. I know I got scared when I saw how much my medication [cost]. if I had to pay out of pocket, I just wouldn’t have it. But there are ways to bring costs down. Because that was kind of a powerful discussion we had in our group, there were things that was mentioned that people did not know.” – Peers LEAD Participant #5*
Learn how to read nutrition labels	*“How to read [nutrition] labels for content … because if you think you’re eating a Lean Cuisine, and you think it’s healthy for you, if you can read the contents, you’ll know that it’s not.” – Peers LEAD Participant #9*
Explore holistic approaches to diabetes management, including alternative medicine and exercise	*“The topics are wonderful. But I think there needs to include like instead of medication, a holistic approach to it…So if, different teas, different herbs, things such as that, if that could be shared, and if it’s really true that it work, that would be good to educate African Americans on without having to take a lot of medication.” – Peers LEAD Participant #1* *“One of the things that was initially touched on in Peers LEAD about fitness and working out and weightlifting and, you know, just being a little bit more active. I wish that would’ve been … focused on a little bit more.” – Peers LEAD Participant #3*

## Data Availability

the data used and/or analyzed during the current study are available from the corresponding author on reasonable request.

## Abbreviations

HLWD: Healthy Living with diabetes
Peers LEAD: Peers Supporting Health Literacy, Self-Efficacy, Self-Advocacy, and Adherence
Peers EXCEL: Peers’ Experience in Communicating and Engaging in Healthy Living
CHW: Community Health Worker
IRB: Institutional Review Board
CFIR: Consolidated Framework for Implementation Research
PI: Principal Investigator
ICTR-CAP: Institute for Clinical and Translational Research-Community Academic Partnership
CRT: Critical Race Theory
